# Gender differences in prevalence and clinical risk factors of suicide attempts in young adults with first-episode drug-naive major depressive disorder

**DOI:** 10.1192/bjo.2023.635

**Published:** 2024-01-05

**Authors:** Quanfeng Zhu, Xiaoe Lang, Xiang-Yang Zhang

**Affiliations:** Affiliated Xiaoshan Hospital, Hangzhou Normal University, Hangzhou, China; Department of Psychiatry, First Hospital/First Clinical Medical College of Shanxi Medical University, Taiyuan, China; CAS Key Laboratory of Mental Health, Institute of Psychology, Beijing, China; and Department of Psychology, University of the Chinese Academy of Sciences, Beijing, China

**Keywords:** First-episode, drug-naive, adolescence, major depressive disorder, suicide attempts

## Abstract

**Background:**

Suicide rates in adolescents with major depressive disorder (MDD) change with age and gender. Early adulthood is an important transitional stage between late adolescence and adulthood, in which an individual's mind gradually matures. However, there are fewer studies on prevalence and variables linked to the suicide attempts of young adults with MDD.

**Aims:**

To explore gender differences in the prevalence and risk factors associated with suicide attempts in young adults with first-episode drug-naive MDD.

**Method:**

The Hamilton Rating Scale for Depression (HRSD), Hamilton Rating Scale for Anxiety (HRSA) and Positive Subscale of the Positive and Negative Syndrome Scale (PANSS) were used to assess depression, anxiety and psychotic symptoms respectively and various biochemical indicators were assessed.

**Results:**

Among 293 young adults with first-episode drug-naive MDD, the prevalence of suicide attempts was 15.45% (19/123) for males and 14.12% (24/170) for females. Males with suicide attempts had higher levels of thyroid-stimulating hormone (TSH) and higher PANSS Positive Subscale scores, whereas females with suicide attempts had higher TSH, serum total cholesterol, fasting blood glucose and diastolic blood pressure levels and higher scores on the HRSD, HRSA, PANSS Positive Subscale (all Bonferroni corrected *P* < 0.05). In males, PANSS Positive Subscale score (*B* = 0.17, *P* = 0.03, OR = 1.19, 95% CI 1.02–1.38) was a risk factor for suicide attempts.

**Conclusions:**

There were significant gender differences in the risk factors for suicide attempts in young adults with first-episode drug-naive MDD.

Major depressive disorder (MDD) is a common mental disease worldwide, and according to rough statistics, perhaps one in five people will be affected by MDD in their lifetime.^1^ Globally, MDD is a major cause of disability and ranks second in terms of disease burden. Its detrimental effects on public health outweigh those of conditions such as diabetes and coronary heart disease.^2,3^ The cause of MDD is very complex and involves a combination of environmental, psychological and genetic factors.^4^ MDD has a wide range of symptoms, and patients are affected by it in many ways, including emotional, cognitive and behavioural.^5^ MDD is not only a disease of adults; on the contrary, studies show that depression is becoming more prevalent in adolescents, with approximately 8−21% of 18-year-olds likely to experience it.^6,7^ Several studies have found that depression has a greater risk of onset in adolescence between the ages of 15 and 18 years, and that gender differences in depression are significantly higher in this age group.^6^ Therefore, adolescence is likely to be a critical period for studying susceptibility to depression.

Numerous previous studies have confirmed disparities between genders in MDD prevalence and clinical manifestations. For example, some meta-analyses report that the prevalence of MDD in females is roughly twice that in males.^8,9^ In addition, some findings confirm that females with MDD also have a higher probability of comorbid anxiety, sadness and somatic symptoms than males.^10,11^ On the other hand, suicidal and addictive behaviours are more common among males with MDD.^12^ The causes of these gender differences are unclear, although they may include genetic factors and sex hormones. These gender differences in MDD symptoms are directly related to clinical treatment decisions; therefore, exploring gender differences in MDD symptoms can help refine clinical treatment.

Depression has long been recognised as an important risk factor for suicide. Suicide risk among patients with MDD is up to 20 times higher than that in healthy people, and 50% of suicides worldwide occur during episodes of depression.^13^ The determinants of suicide risk in adolescence and early adulthood are slightly different from those in adulthood, and much of the current research focuses more on the social factors that influence adolescents’ suicidal behaviours, such as examination pressures, bullying in schools and parental loss as possible suicide attempt risk factors.^14^ It is been shown that mental problems have a major influence on suicide attempts in adolescence and young adulthood.^15^ However, there are few reports related to the factors influencing suicide attempts in people with MDD in early adulthood. Research has revealed disparities between genders in the frequency of teenage suicide attempts. For example, a meta-analysis found that in the adolescent population, females had a higher risk of attempted suicide, whereas males had a higher risk of death by suicide.^16^ There may also be some gender differences in the effect of depression on suicide: the findings of Papadopoulou^17^ illustrate that there is a higher correlation between depression and suicide risk in females compared with males.

Although the results of prior studies have confirmed significant gender differences in prevalence and correlates of adolescent suicide, it is important to note that various psychiatric disorders as well as substance use have a high impact on suicide risk. There has been less research on whether the same gender disparities in the frequency and determinants of attempted suicide exist in young people with MDD after excluding confounders such as other psychiatric disorders and substance use disorders. Our research sought to investigate gender differences in prevalence and clinically relevant factors of suicide attempts among young adults with first-episode drug-naive MDD.

## Method

### Recruiting conditions

The Helsinki Declaration of 1975, as amended in 2008, and the ethical guidelines established by the appropriate national and institutional committees on human experimentation apply to all techniques used in the course of this work. This study was approved by the Ethics Committee of the First Hospital of Shanxi Medical University (no. 2016-Y27).

A total of 293 patients from the out-patient clinics of the First Hospital of Shanxi Medical University were recruited. After being fully informed of purpose and protocol of the study, each recruited participant gave written informed consent.

The inclusion criteria were: (a) aged 18–20 years (‘young adults’) and of the Chinese Han population;^18^ (b) a diagnosis of MDD according to DSM-IV; and (c) never having been treated with antidepressants or antipsychotics. The exclusion criteria were: (a) diagnosis of psychiatric disorders other than MDD; (b) presence of organic brain disease; (3) drug dependence; (4) malignancy or immune dysfunction; (5) currently pregnant.

### Demographic characteristics

Sociodemographic information on each participant was collected by questionnaire, including: age, gender, education, duration of depressive illness and marital status. The height, weight and body mass index (BMI) of each participant were recorded.

### Suicide attempts

Each participant's experience with suicide was recorded through a face-to-face interview. Each participant was asked ‘Have you ever attempted suicide in your life?’ If the answer was ‘yes’, further specific information was recorded, including the number of suicide attempts, the method and the exact date of each attempt.

### Clinical symptom assessment

The Positive and Negative Syndrome Scale (PANSS) Positive Subscale, the 14-item Hamilton Rating Scale for Anxiety (HRSA-14) and the 17-item Hamilton Rating Scale for Depression (HRSD-17) were used to assess participants’ psychotic, anxiety and depressive symptoms. It has been demonstrated that the Chinese versions of these scales are valid and reliable among Chinese populations.^19–21^ To ensure the reliability of the assessment results, both psychiatrists responsible for the assessments attended training in the use of the three scales before assessment. After training, the correlation coefficient between the scores on each scale evaluated by the two psychiatrists was greater than 0.8.

### Measurement of blood parameters

Participants were directed to fast from 20.00 h the night before, and blood pressure (BP) measurement and blood sample collection were completed between 06.00 and 08.00 h the next day. The following biochemical indicators were assessed: thyroid-stimulating hormone (TSH), anti-thyroglobulin antibodies (TgAb), thyroid peroxidase antibodies (TPOAb), free triiodothyronine (FT3), free thyroxine (FT4), fasting blood glucose (FBG), serum total cholesterol, triglyceride, low-density lipoprotein cholesterol (LDL-C) and high-density lipoprotein cholesterol (HDL-C) levels. FBG, total cholesterol, triglycerides, HDL-C and LDL-C were measured using an Abbott ARCHITECT *c*8000 clinical chemistry analyser. FT3, FT4, TSH, TgAb and TPOAb were measured using a Roche cobas 6000 electrochemiluminescence immunoassay analyser. Blood pressure was measured using an Omron HBP-1300 electronic sphygmomanometer.

### Statistical analysis

The Kolmogorov–Smirnov test was used to determine whether continuous variables were normally distributed. The *t*-test was used to assess continuous variables with normal distributions, the Mann–Whitney *U*-test was used to test continuous variables without normal distributions and the chi-squared test was used to test categorical variables. First, we grouped the participants by gender and performed univariate analysis of the above indicators between the two groups to investigate the gender differences in terms of sociodemographic information, clinical symptoms and biochemical indicators. Then, we conducted univariate analysis with suicide attempts as the dependent variable and the other variables as independent variables to analyse the factors associated with suicide attempts. Finally, binary logistic regression analyses with suicide attempt as the dependent variable and variables that differed significantly after univariate analyses as independent variables was conducted to examine the risk variables linked to suicide attempts. All statistical analyses were completed using SPSS 25.0 software for Windows. The statistical results of all univariate analyses were subjected to Bonferroni correction with a corrected *P*-value of 0.05 as the threshold for significance.

## Results

### Patient distribution

Of the 293 participants, 123 were male and 170 were female. Among male participants, 11 were married and 112 were unmarried, the mean age was 18.69 years (s.d. = 0.79), the mean duration of illness was 4.06 months (s.d. = 3.03), the mean BMI was 24.57 (s.d. = 2.420) and 68 (55.28%) were overweight/obese. Among female participants, 13 were married and 157 were unmarried, the mean age was 18.68 years (s.d. = 0.83), the mean duration of illness was 3.88 months (s.d. = 2.63), the mean BMI was 23.82 (s.d. = 1.88) and 80 (47.06%) were overweight/obese. There were no significant differences between male and female participants in these sociodemographic factors, clinical symptom scores and levels of biochemical indicators (all *P* > 0.05) (Table 1).

**Table 1:** Sociodemographic characteristics, clinical symptom scores and levels of biochemical indicators of 293 young adults with first-episode drug-naive major depressive disorder

Variable^a^	Males (*n* = 123)	Females (*n* = 170)	*F*/χ^2^/*Z*	Bonferroni-corrected *P*^b^
Age, years: median (P_25%_, P_75%_)	18 (18, 19)	18 (18, 19)	−0.32	1
Duration of illness, months: median (P_25%_, P_75%_)	3 (2, 5)	3 (2, 5)	−0.03	1
Education			−1.24	1
Junior high school, *n* (%)	7 (5.7%)	2 (1.2%)		
High school, *n* (%)	89 (72.4%)	126 (74.1%)		
University degree, *n* (%)	26 (21.1%)	37 (21.8%)		
Master's degree, *n* (%)	1 (0.8%)	5 (2.9%)		
Marital status			0.16	1
Single, *n* (%)	112 (91.1%)	157 (92.4%)		
Married, *n* (%)	11 (8.9%)	13 (7.6%)		
HRSD score, median (P_25%_, P_75%_)	30 (28, 32)	30 (28, 32)	−0.24	1
HRSA score, median (P_25%_, P_75%_)	21 (18, 22)	21 (18, 23)	−0.68	1
PANSS Positive Subscale score, median (P_25%_, P_75%_)	7 (7, 7)	7 (7, 7)	−0.67	1
BMI, kg/m^2^: median (P_25%_, P_75%_)	24.15 (23.18, 26.12)	23.88 (22.49, 25.23)	−2.33	0.39
Systolic BP, mmHg: mean (s.d.)	109.95 (8.97)	109.34 (9.89)	1.17	1
Diastolic BP, mmHg: median (P_25%_, P_75%_)	70 (68, 75)	72 (68, 76)	−0.61	1
Total cholesterol, mmol/L: mean (s.d.)	5.14 (1.15)	5.01 (1.20)	0.05	1
Triglycerides, mmol/L: median (P_25%_, P_75%_)	1.99 (1.31, 2.82)	1.95 (1.36, 2.83)	−0.07	1
HDL-C, mmol/L: median (P_25%_, P_75%_)	1.26 (1.01, 1.41)	1.26 (1.10, 1.45)	−0.91	1
LDL-C, mmol/L: mean (s.d.)	2.88 (0.92)	2.87 (0.94)	0.07	1
FBG, mmol/L: median (P_25%_, P_75%_)	5.19 (4.80, 5.67)	5.29 (4.92, 5.78)	−1.95	1
TSH, μIU/mL: median (P_25%_, P_75%_)	4.63 (3.22, 6.62)	4.24 (2.83, 6.23)	−1.12	1
FT3, pmol/L: mean (s.d.)	5.02 (0.77)	4.93 (0.75)	0.53	1
FT4, pmol/L: median (P_25%_, P_75%_)	17.21 (14.71, 19.55)	16.53 (14.48, 18.13)	−1.42	1
TgAb, IU/L: median (P_25%_, P_75%_)	18.90 (13.26, 25.44)	18.99 (13.32, 30.88)	−0.38	1
TPOAb, IU/L: median (P_25%_, P_75%_)	15.70 (12.29, 27.24)	18.65 (11.63, 32.55)	−0.90	1

P_25%_, 25th percentile; P_75%_, 75th percentile; HRSD, Hamilton Rating Scale for Depression; HRSA, Hamilton Rating Scale for Anxiety; PANSS, Positive and Negative Syndrome Scale; BMI, body mass index; BP, blood pressure; HDL-C, high-density lipoprotein cholesterol; LDL-C, low-density lipoprotein cholesterol; FBG, fasting blood glucose; TSH, thyroid-stimulating hormone; FT3, free triiodothyronine; FT4, free thyroxine; TgAb, anti-thyroglobulin antibodies; TPOAb, thyroid peroxidase antibodies.

a. Continuous variables conforming to normal distribution: mean (s.d.); continuous variables that do not conform to normal distribution: median (25th percentile, 75th percentile).

b. Bonferroni-corrected *P* > 1 is shown as *P* = 1.

### Prevalence of suicide attempts

The prevalence of attempted suicide was 15.45% (19/123) among males and 14.12% (24/170) among females. The gender difference was non-significant (*P* > 0.05).

### Analysis of factors associated with suicide attempts

As shown in Tables 2 and 3, males with suicide attempts had higher TSH levels and PANSS Positive Subscale scores (both *P* < 0.05), whereas females with suicide attempts had higher TSH, total cholesterol, FBG, diastolic BP levels and higher PANSS Positive Subscale, HRSD and HRSA scores (all *P* < 0.05).

**Table 2:** Sociodemographic and clinical characteristics of male participants with and without suicide attempts

Variable^a^	Participants with suicide attempts (*n* = 19)	Participants without suicide attempts (*n* = 104)	*F*/χ^2^/*Z*	Bonferroni-corrected *P*^b^	Effect sizes, Cohen's *d*/φ
Age, years: median (P_25%_, P_75%_)	19 (18, 20)	18 (18, 19)	−0.91	1	0.22
Duration of illness, months: median (P_25%_, P_75%_)	3 (2, 6)	3 (2, 5)	−0.54	1	0.25
Education			−0.36	1	0.05
Junior high school, *n* (%)	0	7 (6.7%)			
High school, *n* (%)	15 (78.9%)	74 (71.2%)			
University degree, *n* (%)	4 (21.1%)	22 (21.2%)			
Master's degree, *n* (%)	0	1 (0.9%)			
Marital status			0	1	0
Single, *n* (%)	17 (89.5%)	95 (91.3%)			
Married, *n* (%)	2 (10.5%)	9 (8.7%)			
HRSD score, median (P_25%_, P_75%_)	32 (29, 34)	29 (28, 32)	−2.91	0.07	0.74
HRSA score, median (P_25%_, P_75%_)	21 (21, 23)	20 (18, 22)	−2.79	0.11	0.77
PANSS Positive Subscale score, median (P_25%_, P_75%_)	7 (7, 12)	7 (7, 7)	−3.23	0.02	0.78
BMI, kg/m^2^: median (P_25%_, P_75%_)	23.74 (22.14, 25.12)	24.20 (23.23, 26.26)	−1.63	1	0.14
Systolic BP, mmHg: mean (s.d.)	114.37 (8.60)	109.14 (8.84)	0.20	0.38	0.60
Diastolic BP, mmHg: median (P_25%_, P_75%_)	72 (70, 77)	70 (67.25, 75)	−1.37	1	0.36
Total cholesterol, mmol/L: mean (s.d.)	5.58 (1.33)	5.06 (1.10)	1.13	1	0.42
Triglycerides, mmol/L	2.00 (1.01)	2.21 (1.03)	0.20	1	0.21
HDL-C, mmol/L: median (P_25%_, P_75%_)	1.12 (0.86, 1.28)	1.27 (1.08, 1.41)	−1.95	1	0.40
LDL-C, mmol/L: median (P_25%_, P_75%_)	3.30 (2.72, 4.20)	2.66 (2.23, 3.36)	−1.98	0.94	0.45
FBG, mmol/L: mean (s.d.)	5.40 (0.54)	5.23 (0.63)	0.67	1	0.29
TSH, μIU/mL: mean (s.d.)	6.44 (2.55)	4.62 (2.32)	0.01	0.047	0.75
FT3, pmol/L: mean (s.d.)	4.91 (0.82)	5.04 (0.76)	0.69	1	0.16
FT4, pmol/L: mean (s.d.)	17.89 (3.04)	17.02 (3.32)	0.77	1	0.27
TgAb, IU/L: median (P_25%_, P_75%_)	16.18 (14.11, 123.60)	18.91 (13.21, 24.43)	−0.77	1	0.06
TPOAb, IU/L: median (P_25%_, P_75%_)	21.21 (15.25, 134.20)	15.26 (12.25, 22.85)	−2.47	0.27	0.55

P_25%_, 25th percentile; P_75%_, 75th percentile; HRSD, Hamilton Rating Scale for Depression; HRSA, Hamilton Rating Scale for Anxiety; PANSS, Positive and Negative Syndrome Scale; BMI, body mass index; BP, blood pressure; HDL-C, high-density lipoprotein cholesterol; LDL-C, low-density lipoprotein cholesterol; FBG, fasting blood glucose; TSH, thyroid-stimulating hormone; FT3, free triiodothyronine; FT4, free thyroxine; TgAb, anti-thyroglobulin antibodies; TPOAb, thyroid peroxidase antibodies.

a. Continuous variables conforming to normal distribution: mean (s.d.); continuous variables that do not conform to normal distribution: median (25th percentile, 75th percentile).

b. Bonferroni-corrected *P* > 1 is shown as *P* = 1.

**Table 3:** Sociodemographic and clinical characteristics of female participants with and without suicide attempts

Variable	Participants with suicide attempts (*n* = 24)	Participants without suicide attempts (*n* = 146)	*F*/χ^2^/*Z*	Bonferroni-corrected P	Effect sizes, Cohen's *d*/φ
Age, years: median (P_25%_, P_75%_)	18.5 (18, 20)	18 (18, 19)	−0.70	1	0.16
Duration of illness, months: median (P_25%_, P_75%_)	3.25 (2.13, 6)	3 (2, 5)	−0.54	1	0.11
Education			−0.20	1	0.03
Junior high school, *n* (%)	1 (4.2%)	1 (0.7%)			
High school, *n* (%)	17 (70.8%)	109 (74.7%)			
University degree, *n* (%)	5 (20.8%)	32 (21.9%)			
Master's degree, *n* (%)	1 (4.2%)	4 (2.7%)			
Marital status			1.23	1	0.09
Single, *n* (%)	24 (100%)	133 (91.1%)			
Married, *n* (%)	0	13 (8.9%)			
HRSD score, median (P_25%_, P_75%_)	33 (31, 35)	30 (27, 32)	−4.81	<0.001	1.23
HRSA score, median (P_25%_, P_75%_)	24 (21, 26.75)	20 (18, 22)	−4.03	0.001	0.99
PANSS Positive Subscale score, median (P_25%_, P_75%_)	8.5 (7, 21)	7 (7, 7)	−3.76	0.003	0.80
BMI, kg/m^2^: mean (s.d.)	23.33 (2.36)	23.90 (1.79)	1.12	1	0.28
Systolic BP, mmHg: mean (s.d.)	116.67 (12.95)	108.13 (8.78)	5.94	0.09	0.79
Diastolic BP, mmHg: median (P_25%_, P_75%_)	76.50 (70.50, 80.75)	70.5 (68, 75)	−3.46	0.01	0.82
Total cholesterol, mmol/L: mean (s.d.)	5.96 (1.18)	4.85 (1.13)	0.42	<0.001	0.97
Triglycerides, mmol/L: median (P_25%_, P_75%_)	1.88 (1.34, 2.94)	2 (1.36, 2.81)	−0.11	1	0.001
HDL-C, mmol/L: mean (s.d.)	1.13 (0.29)	1.28 (0.25)	1.18	0.12	0.58
LDL-C, mmol/L: mean (s.d.)	3.32 (1.05)	2.80 (0.91)	0.52	0.25	0.53
FBG, mmol/L: median (P_25%_, P_75%_)	5.89 (5.39, 6.38)	5.26 (4.85, 5.68)	−3.76	0.003	0.82
TSH, μIU/mL: mean (s.d.)	7.29 (3.11)	4.22 (2.27)	7.21	0.002	1.14
FT3, pmol/L: mean (s.d.)	4.92 (0.81)	4.93 (0.74)	0.50	1	0.02
FT4, pmol/L: median (P_25%_, P_75%_)	16.36 (15.20, 17.51)	16.57 (14.30, 18.21)	−0.07	1	0.04
TgAb, IU/L: median (P_25%_, P_75%_)	25.72 (15.88, 175.83)	17.83 (13.16, 28.40)	−2.27	0.46	0.09
TPOAb, IU/L: median (P_25%_, P_75%_)	27.50 (12.50, 126.71)	17.75 (11.61, 29.06)	−1.79	1	0.46

P_25%_, 25th percentile; P_75%_, 75th percentile; HRSD, Hamilton Rating Scale for Depression; HRSA, Hamilton Rating Scale for Anxiety; PANSS, Positive and Negative Syndrome Scale; BMI, body mass index; BP, blood pressure; HDL-C, high-density lipoprotein cholesterol; LDL-C, low-density lipoprotein cholesterol; FBG, fasting blood glucose; TSH, thyroid-stimulating hormone; FT3, free triiodothyronine; FT4, free thyroxine; TgAb, anti-thyroglobulin antibodies; TPOAb, thyroid peroxidase antibodies.

a. Continuous variables conforming to normal distribution: mean (s.d.); continuous variables that do not conform to normal distribution: median (25th percentile, 75th percentile).

b. Bonferroni-corrected *P* > 1 is shown as *P* = 1.

After taking the variables with significant differences as covariates in binary logistic regression analysis, the results suggested that, among males, the factors that influenced suicide attempts were TSH level (*B* = 0.21, *P* = 0.08, OR = 1.24, 95% CI 0.98–1.57) and PANSS Positive Subscale score (*B* = 0.17, *P* = 0.03, OR = 1.19, 95% CI 1.02–1.38). Among female patients, the factors that influenced suicide attempts were TSH level (*B* = 0.14, *P* = 0.35, OR = 1.15, 95% CI 0.86–1.52), total cholesterol level (*B* = 0.09, *P* = 0.78, OR = 1.09, 95% CI 0.59–2.00), diastolic BP (*B* = 0.04, *P* = 0.47, OR = 1.04, 95% CI 0.93–1.17), HRSD score (*B* = 0.21, *P* = 0.15, OR = 1.24, 95% CI 0.92–1.65), HRSA score (*B* = 0.15, *P* = 0.20, OR = 1.16, 95% CI 0.93–1.46), PANSS Positive Subscale score (*B* = −0.02, *P* = 0.76, OR = 0.98, 95% CI 0.87–1.11) and FBG level (*B* = 0.60, *P* = 0.19, OR = 1.82, 95% CI 0.75–4.42).

## Discussion

The primary conclusions drawn from the study are: (a) the prevalence of suicide attempts among young adults with first-episode drug-naive MDD was approximately 15%, with no significant gender differences; (b) among participants with suicide attempts, males had significantly higher TSH levels and PANSS Positive Subscale scores, and females had higher TSH, total cholesterol, FBG and diastolic BP levels, as well as higher HRSD, HRSA and PANSS Positive Subscale scores; and (c) positive symptoms of schizophrenia were a risk factor for suicide attempts among males.

### Interpretation of findings and comparison with the literature

It has been reported that suicide attempts during adolescence are far more common among females than among males and they also happen at a much younger age among females.^22^ In the adolescent population, this gender difference in incidence of suicidal behaviour is not constant, but changes with age. The incidence of suicide attempts among adolescent females may peak with age in mid-adolescence and decrease in late adolescence, whereas the incidence among adolescent males may continue to increase with age until early adulthood.^23^ We believe that emotional and behavioural problems, psychiatric disorders and substance misuse all contribute to these gender differences in suicidal behaviour. We found no disparities between genders in the frequency of attempted suicide in our sample of individuals with MDD in late adolescence after excluding confounding factors of mental illness and medications.

HRSD and HRSA scores are commonly used to reflect the intensity of depressive symptoms and to predict the occurrence of suicide attempts in MDD.^24^ In our study, the correlation between higher HRSD and HRSA scores and suicide attempts was found only in females, and it is unclear whether this gender difference is due to sample heterogeneity or is widespread, and further studies with larger samples are needed.

Suicidal behaviour may be driven by positive psychotic symptoms. Compared with individuals with MDD without psychotic symptoms, those with psychotic symptoms are more than twice as likely to attempt suicide.^25^ Positive symptoms can directly or indirectly affect suicide risk. For example, delusions and hallucinations can directly affect suicidal behaviour, whereas other positive symptoms may mediate the relationship with suicidal ideation by affecting cognitive function and insight.^26–28^ The results of our study support these ideas, as suicide attempts were significantly associated with higher PANSS Positive Subscale scores in our sample of young adults. However, regression analysis revealed that the PANSS Positive Subscale score was substantially associated with suicide attempts only in males, whereas in females other factors also influenced suicide attempts, so that the independent effect of positive symptoms on suicide attempts was no longer statistically significant. This seems to suggest that in young adults with MDD positive symptoms are a stronger driver of suicide attempts in males than in females. This is one of the primary conclusions of our research, which suggests that more attention should be paid to positive symptoms in males with MDD in early adulthood, because positive symptoms may affect the prevalence of suicide attempts in this group, in addition to causing aggressive behaviour.

Prior research has found a connection between TSH levels and suicide. However, sample heterogeneity as well as other confounding factors, such as different environments, stages of illness, psychiatric comorbidities and antipsychotics and antidepressants, have led to inconsistent findings. For example, Berlin et al found that people with MDD who attempted suicidal tended to have higher TSH levels.^29^ However, the opposite result was reported by Peng et al, who found significantly lower TSH levels in people with MDD who made suicide attempts.^30^ According to our research, there was a substantial correlation between suicide attempts and higher TSH levels. Thyroid diseases are considered to have a strong correlation with symptoms of depression, anxiety and psychosis. In addition, thyroid hormones may have effects on suicidal behaviour through neurotransmitters such as serotonin and noradrenaline.^31,32^ Although using thyroid hormone levels as a predictor of suicide risk should be cautious, it still provides data to support subsequent studies.

There is no universally accepted conclusion on the correlation between total cholesterol or other lipid levels and suicide in people with MDD and psychotic symptoms. There are many reasons for these differences in results, and in addition to sample heterogeneity, racial differences in lipid profiles and lipoprotein lipase activity in different studies may also be an important reason.^33^ In addition, the effect of different antipsychotics and antidepressants on blood lipids was an important confounding factor in the differences in study results. In our study, we observed significantly higher levels of total cholesterol in females with suicide attempts but not in males. The possible higher sensitivity of females to diet-induced changes in central serotonin function may have contributed to gender differences in our study results.^34^

Abnormal glucose metabolism is thought to accelerate the process of MDD, and people with MDD are more prone to having impaired glucose metabolism than the healthy population.^35,36^ Among people with MDD, abnormal glucose metabolism may influence suicide attempts through more severe symptoms of depression and anxiety. High blood pressure may be indicative of a greater risk of cardiovascular disease. Abnormal glucose metabolism and hypertension cause patients to exhibit greater despair and suicidal ideation, which may account for the correlation between these factors and suicide attempts.^37^ In the present study, we found significant correlations between higher levels of blood glucose and blood pressure and suicide attempts only in females. As is often shown, gender differences exist in the occurrence and development of hypertension and diabetes, as well as in the risk of cardiovascular disease they cause, which may result from biological gender differences in genetic susceptibility, gene expression, hormone levels, etc.^38^ We believe that the associated gender difference in our findings may also be related to these factors. Interestingly, the higher blood glucose and blood pressure levels in the group at high risk of suicide attempts in our study remained within normal values and did not meet the criteria for hyperglycaemia and hypertension, and therefore we cannot explain their correlation with suicide attempts in terms of the effects of abnormal glucose metabolism or hypertension. Such results seem to signal the importance of controlling blood pressure and blood glucose levels in people with MDD. A previous study revealed that controlling blood glucose and blood pressure can reduce all-cause mortality in obese populations, especially in Chinese populations.^39^ Our findings illustrate that even when blood pressure and glucose are at normal levels, patients still seem to benefit from keeping them in a reasonably low range. Of course, this needs to be verified with more research.

### Limitations

A few limitations apply to our study. First, as this was a cross-sectional study, we were unable to further explore the causal relationship between suicide attempts and associated factors. Second, we did not set up a healthy control group to investigate the risk factors for MDD. Third, all participants in this study were from the same hospital out-patient department, which may make the study results somewhat limited. Fourth, information on participants’ religious beliefs was not collected in this study, and religious ideology in some areas may be one of the influencing factors for suicide attempts. In addition, information on suicide attempts in this study was obtained from participants’ self-reports, and since some of the information provided by participants was not verified by family members, there is a possibility that participants reported inaccurate information. Fifth, the sample size of this study was not large enough because we limited the age range of early adults too narrowly, which will have resulted in some limitations in the representativeness of our findings. We will collect more samples in a future study.

### Implications

Our findings may inform clinicians in their treatment of MDD in young adults and may also provide a reference for subsequent studies on the risk of suicide in MDD.

## Data Availability

Raw data from this study are available from the corresponding author, X.-Y.Z., on reasonable request.

## References

[1] Block SG, Nemeroff CB. Emerging antidepressants to treat major depressive disorder. Asian J Psychiatr 2014; 12: 7–16. 10.1016/j.ajp.2014.09.001 25277330

[2] Ménard C, Hodes GE, Russo SJ. Pathogenesis of depression: insights from human and rodent studies. Neuroscience 2016; 321: 138–62. 26037806 10.1016/j.neuroscience.2015.05.053 PMC4664582

[3] Smith K. Mental health: a world of depression. Nature 2014; 515: 181. 25391942 10.1038/515180a

[4] Uchida S, Yamagata H, Seki T, Watanabe Y. Epigenetic mechanisms of major depression: targeting neuronal plasticity. Psychiatry Clin Neurosci 2018; 72: 212–27. 29154458 10.1111/pcn.12621

[5] Culpepper L, Lam RW, McIntyre RS. Cognitive impairment in patients with depression: awareness, assessment, and management. J Clin Psychiatry 2017; 78: 1383–94. 29345866 10.4088/JCP.tk16043ah5c

[6] Hankin BL, Abramson LY, Moffitt TE, Silva PA, McGee R, Angell KE. Development of depression from preadolescence to young adulthood: emerging gender differences in a 10-year longitudinal study. J Abnorm Psychol 1998; 107: 128–40. 9505045 10.1037//0021-843x.107.1.128

[7] Cheung AH, Dewa CS. Canadian community health survey: major depressive disorder and suicidality in adolescents. Healthc Policy 2006; 2: 76–89. 19305706 PMC2585433

[8] Porras-Segovia A, Valmisa E, Gutiérrez B, Ruiz I, Rodríguez-Barranco M, Cervilla J. Prevalence and correlates of major depression in Granada, Spain: results from the GranadΣp study. Int J Soc Psychiatry 2018; 64: 450–8. 29843555 10.1177/0020764018771405

[9] Salk RH, Hyde JS, Abramson LY. Gender differences in depression in representative national samples: meta-analyses of diagnoses and symptoms. Psychol Bull 2017; 143: 783–822. 28447828 10.1037/bul0000102 PMC5532074

[10] Labonté B, Engmann O, Purushothaman I, Menard C, Wang J, Tan C, et al. Sex-specific transcriptional signatures in human depression. Nat Med 2017; 23: 1102–11. 28825715 10.1038/nm.4386 PMC5734943

[11] Martin LA, Neighbors HW, Griffith DM. The experience of symptoms of depression in men vs women: analysis of the national comorbidity survey replication. JAMA Psychiatry 2013; 70: 1100–6. 23986338 10.1001/jamapsychiatry.2013.1985

[12] Shi P, Yang A, Zhao Q, Chen Z, Ren X, Dai Q. A hypothesis of gender differences in self-reporting symptom of depression: implications to solve under-diagnosis and under-treatment of depression in males. Front Psychiatry 2021; 12: 589687. 34759845 10.3389/fpsyt.2021.589687 PMC8572815

[13] Chesney E, Goodwin GM, Fazel S. Risks of all-cause and suicide mortality in mental disorders: a meta-review. World Psychiatry 2014; 13: 153–60. 24890068 10.1002/wps.20128 PMC4102288

[14] Rodway C, Tham S-G, Ibrahim S, Turnbull P, Windfuhr K, Shaw J, et al. Suicide in children and young people in England: a consecutive case series. Lancet Psychiatry 2016; 3: 751–9. 27236279 10.1016/S2215-0366(16)30094-3

[15] Shain BN. Youth suicide: the first suicide attempt. J Am Acad Child Adolesc Psychiatry 2018; 57: 730–2. 30274647 10.1016/j.jaac.2018.05.022

[16] Miranda-Mendizabal A, Castellví P, Parés-Badell O, Alayo I, Almenara J, Alonso I, et al. Gender differences in suicidal behavior in adolescents and young adults: systematic review and meta-analysis of longitudinal studies. Int J Public Health 2019; 64: 265–83. 30635683 10.1007/s00038-018-1196-1 PMC6439147

[17] Papadopoulou A, Efstathiou V, Christodoulou C, Gournellis R, Papageorgiou C, Douzenis A, et al. Psychiatric diagnosis, gender, aggression, and mode of attempt in patients with single versus repeated suicide attempts. Psychiatry Res 2020; 284: 112747 (10.1016/j.psychres.2020.112747). 31927168

[18] Dimler LM, Natsuaki MN. Trajectories of violent and nonviolent behaviors from adolescence to early adulthood: does early puberty matter, and, if so, how long? J Adolesc Health 2021; 68: 523–31. 32928642 10.1016/j.jadohealth.2020.06.034

[19] Kay SR, Fiszbein A, Opler LA. The Positive and Negative Syndrome Scale (PANSS) for schizophrenia. Schizophr Bull 1987; 13: 261–76. 3616518 10.1093/schbul/13.2.261

[20] Lin GX. [Uses of HAMA the rating scale in neurosis]. Zhonghua Shen Jing Jing Shen Ke Za Zhi 1986; 19: 342–4. 3582026

[21] Sun XY, Li YX, Yu CQ, Li LM. [Reliability and validity of depression scales of Chinese version: a systematic review]. Zhonghua Liu Xing Bing Xue Za Zhi 2017; 38: 110–6. 28100388 10.3760/cma.j.issn.0254-6450.2017.01.021

[22] Wunderlich U, Bronisch T, Wittchen HU, Carter R. Gender differences in adolescents and young adults with suicidal behaviour. Acta Psychiatr Scand 2001; 104: 332–9. 11722313 10.1034/j.1600-0447.2001.00432.x

[23] Boeninger DK, Masyn KE, Feldman BJ, Conger RD. Sex differences in developmental trends of suicide ideation, plans, and attempts among European American adolescents. Suicide Life Threat Behav 2010; 40: 451–64. 21034208 10.1521/suli.2010.40.5.451 PMC2995258

[24] Liu W, Wu Z, Sun M, Zhang S, Yuan J, Zhu D, et al. Association between fasting blood glucose and thyroid stimulating hormones and suicidal tendency and disease severity in patients with major depressive disorder. Bosn J Basic Med Sci 2022; 22: 635–42. 35238287 10.17305/bjbms.2021.6754 PMC9392982

[25] Gournellis R, Tournikioti K, Touloumi G, Thomadakis C, Michalopoulou PG, Christodoulou C, et al. Psychotic (delusional) depression and suicidal attempts: a systematic review and meta-analysis. Acta Psychiatr Scand 2018; 137: 18–29. 29178463 10.1111/acps.12826

[26] Kjelby E, Sinkeviciute I, Gjestad R, Kroken RA, Løberg EM, Jørgensen HA, et al. Suicidality in schizophrenia spectrum disorders: the relationship to hallucinations and persecutory delusions. Eur Psychiatry 2015; 30: 830–6. 26443050 10.1016/j.eurpsy.2015.07.003

[27] Villa J, Choi J, Kangas JL, Kaufmann CN, Harvey PD, Depp CA. Associations of suicidality with cognitive ability and cognitive insight in outpatients with schizophrenia. Schizophr Res 2018; 192: 340–4. 28655480 10.1016/j.schres.2017.06.013 PMC5742308

[28] Bornheimer LA, Wojtalik JA, Li J, Cobia D, Smith MJ. Suicidal ideation in first-episode psychosis: considerations for depression, positive symptoms, clinical insight, and cognition. Schizophr Res 2021; 228: 298–304. 33493778 10.1016/j.schres.2020.12.025 PMC7987901

[29] Berlin I, Payan C, Corruble E, Puech AJ. Serum thyroid-stimulating-hormone concentration as an index of severity of major depression. Int J Neuropsychopharmacol 1999; 2: 105–10. 11281977 10.1017/S146114579900139X

[30] Peng R, Dai W, Li Y. Low serum free thyroxine level is correlated with lipid profile in depressive patients with suicide attempt. Psychiatry Res 2018; 266: 111–5. 29859497 10.1016/j.psychres.2018.05.059

[31] Duntas LH, Maillis A. Hypothyroidism and depression: salient aspects of pathogenesis and management. Minerva Endocrinol 2013; 38: 365–77. 24285104

[32] Krishnan V, Nestler EJ. Linking molecules to mood: new insight into the biology of depression. Am J Psychiatry 2010; 167: 1305–20. 20843874 10.1176/appi.ajp.2009.10030434 PMC3031089

[33] Lee H-J, Kim Y-K. Serum lipid levels and suicide attempts. Acta Psychiatr Scand 2003; 108: 215–21. 12890277 10.1034/j.1600-0447.2003.00115.x

[34] Zhang J, McKeown RE, Hussey JR, Thompson SJ, Woods JR, Ainsworth BE. Low HDL cholesterol is associated with suicide attempt among young healthy women: the Third National Health and Nutrition Examination Survey. J Affect Disord 2005; 89: 25–33. 16263178 10.1016/j.jad.2005.05.021

[35] De Hert M, Cohen D, Bobes J, Cetkovich-Bakmas M, Leucht S, Ndetei DM, et al. Physical illness in patients with severe mental disorders. II. Barriers to care, monitoring and treatment guidelines, plus recommendations at the system and individual level. World Psychiatry 2011; 10: 138–51. 21633691 10.1002/j.2051-5545.2011.tb00036.x PMC3104888

[36] Detka J, Kurek A, Basta-Kaim A, Kubera M, Lasoń W, Budziszewska B. Elevated brain glucose and glycogen concentrations in an animal model of depression. Neuroendocrinology 2014; 100: 178–90. 25300940 10.1159/000368607

[37] Igwe MN, Uwakwe R, Ahanotu CA, Onyeama GM, Bakare MO, Ndukuba AC. Factors associated with depression and suicide among patients with diabetes mellitus and essential hypertension in a Nigerian teaching hospital. Afr Health Sci 2013; 13: 68–77. 23658570 10.4314/ahs.v13i1.10 PMC3645089

[38] Regensteiner JG, Reusch JEB. Sex differences in cardiovascular consequences of hypertension, obesity, and diabetes: JACC focus seminar 4/7. J Am Coll Cardiol 2022; 79: 1492–505. 35422246 10.1016/j.jacc.2022.02.010 PMC9503760

[39] Huang Q, Zou X, Gao P, Han X, Zhou X, Ji L. How does obesity affect mortality through blood pressure and blood glucose in Chinese and US citizens? Insights from a causal mediation analysis of two large cohorts. J Glob Health 2023; 13: 04032. 37022778 10.7189/jogh.13.04032 PMC10078858

